# Odor characterization of the poultry red mite (*Dermanyssus gallinae*) for identification of volatile biomarkers of infestation across multiple commercial laying hen systems

**DOI:** 10.1016/j.psj.2025.105101

**Published:** 2025-03-25

**Authors:** Pascalle J.M. Deenekamp, Iram Gladan, Francisca C. Velkers, Mirlin P. Spaninks, Joris Meurs, Simona M. Cristescu

**Affiliations:** a Life Science Trace Detection Laboratory, Department of Analytical Chemistry, Institute for Molecules & Materials, Radboud University; b Department of Population Health Sciences, Faculty of Veterinary Medicine, Utrecht University, Yalelaan 7, 3584 CL Utrecht, the Netherlands

**Keywords:** Poultry red mite, Laying hen, Volatile organic compound, TD-GC-MS, Odor characterization

## Abstract

Infestations by the poultry red mite (PRM) (*Dermanyssus gallinae)* in laying hen farms can have serious adverse effects on animal health and welfare, and lead to increased egg production costs. Early detection of the PRM is paramount to mitigate its negative impact and for effective pest control. However, current detection methods are labor-intensive and time-consuming. As poultry experts have reported that the presence of PRM is accompanied by a specific scent, opportunities lie in using odor-based detection methods. These methods may offer a fast and reliable alternative for identifying PRM infestations in early stages. To date, however, there is a lack of data on the odor profile of PRM as it has not been characterized. Therefore, in this study, the first step is taken towards determining the composition of the emitted scent of PRM. Samples of PRM and litter were collected across multiple commercial laying hen farms and subjected to TD-GC-MS analysis with subsequent multivariate analysis. Five highly specific volatile organic compound (VOC) targets were identified (1-vinyl-aziridine, 1H-pyrrole, 1-octen-3-one, heptanal and octanal), independent of housing type, feed and farm management. Although the metabolic origin of these VOCs could not be determined in this study, the odor character of several of these VOCs (1-octen-3-one, heptanal and octanal) matches the poultry experts’ description. Furthermore, the specificity of all identified VOC targets to PRM samples make them highly interesting potential targets for odor-based detection of PRM infestation.

## Introduction

The poultry red mite (**PRM**) *Dermanyssus gallinae* (De Geer 1778) is a hematophagous ectoparasite that poses a persisting and major challenge to the global egg production industry with an estimated annual economic impact exceeding €230 million across Europe (Brännström et al., 2008; Sparagano et al., 2009; Sigognault Flochlay et al., 2017; Brauneis et al., 2017). The PRM's blood feeding has detrimental health effects on the hens, including stress, feather pecking, a higher susceptibility to diseases, anemia and even death (Pritchard et al., 2015; Lammers et al., 2017; Oh et al., 2020). Infestations therefore lead to increased financial expenditure due to higher feed and water consumption, higher mortality, decreased egg quality and production, as well as the cost of necessary pest control strategies (Sparagano et al., 2009). Further, studies have indicated that the PRM may play a role as a vector in several human and animal diseases such as encephalitis, fowl poxvirus and *Salmonella* infections (Moro et al., 2005; Sparagano et al., 2014; Schiavone et al., 2022).

Early detection of PRM infestations is important to mitigate these adverse effects but is challenging for several reasons. The PRM progresses through various developmental stages, from egg through larva, protonymph, deutonymph to adult, in only 1-2 weeks under favorable conditions. Further, as adult females lay on average 4-8 eggs (Sparagano et al., 2014), the PRM population grows rapidly when no control measures are in place. Current monitoring methods rely mainly on visual detection of an incipient infestation, which is challenging as the PRM hide in inaccessible refuges in the poultry house structures during the day and only leave them at night to feed (Mul et al., 2015). Early visual detection therefore requires thorough inspection of the aviary system. Another method for early detection involves trapping the PRM onto cardboard paper traps with subsequent counting or weighing (Lammers et al., 2017). However, both visual and trapping methods are time-consuming and labor-intensive, adding onto the poultry flock management workload. If an infestation is not detected in an early stage, pest management strategies are often less effective and more adverse economic and welfare effects will occur (Oh et al., 2020).

A potential method for early detection of PRM lies in the specific odor associated with an infestation. According to poultry experts, PRM presence is characterized by a distinct scent that has been described as a “sweetish, musty and metallic” scent (P. van der Laar & R. Bronneberg, personal communication, 16 August 2023). To date, the origin and composition of this odor have not been identified. The scent could originate from the host (e.g. kairomones), the PRM themselves (e.g. pheromones), or a combination of these two (Pritchard et al., 2015). An odor-based detection method could therefore provide a fast and reliable alternative for detecting poultry red mite infestations in early stages.

Odor-based detection of pests and diseases in livestock and agricultural farming has been researched before, with several examples of early disease detection in the poultry industry (Garner et al., 2008; Grilli et al., 2018; Borgonovo et al., 2020; Golden et al., 2021). Such studies have shown that the (changes in) volatile organic compounds (**VOC**s) released during disease or infestation can serve as biomarkers. For other mite species or pest infestations, VOCs have been used before, e.g. in detection of grain pests such as *Tribolium castaneum* and *Sitophilus granarius* in wheat (Cai et al., 2022).

Here, the first step is taken towards characterization of the odor emitted by PRM. Samples were collected from different laying hen housing systems and compared against litter, which is a major contributor to the odor composition of poultry house air (Dunlop et al., 2016). This background odor in poultry farms can vary widely due to factors including, but not limited to, diurnal changes in poultry house climate, bird activity, the type of litter or feed, and farm management practices (Dunlop et al., 2016). The aim of this study is to identify PRM-specific VOCs that are common across housing systems and can be used for early detection and PRM population monitoring.

## Materials and methods

### On-site Sample Collection

Six laying hen houses across 4 Dutch commercial laying hen farms were included in this field study. The farms were visited between 12 October 2023 and 25 May 2024, between 09.00 h and 12 .00h. PRM population was initially visually evaluated by using the Mite Monitoring Scoring (MMS) system (Cox et al., 2009) and then quantified by AVIVET® poultry red mite traps (Lammers et al., 2017). The traps were placed in the houses for 48 h and retrieved from the poultry house during PRM and litter sample collection. The number of traps and placement in the poultry houses was based on the supplier's recommendation and mite monitoring protocols for laying hens (Decru et al., 2020; Nijs and Sleeckx, 2023). In houses with more than 25,000 chickens, 20 traps were placed, and 10 in houses with less than 25,000 chickens. The traps were evenly distributed throughout the house and placed near the hens’ resting places at night, along paths PRM likely travel to feed on the birds. After collection, the traps were individually sealed in small plastic bags, stored at −18 to −20°C, and processed for quantification by weighing the contents as described previously (Lammers et al. 2017). The housing characteristics of the visited farms are summarized in Table 1.

**Table 1:** Housing characteristics of visited poultry houses on laying hen farms for poultry red mite collection.

	Sampling location^1^					
	A-I	A-II	B-I	B-II	C-I	C-II
Farm Type^2^	Free Range	Free Range	Barn	Barn	Organic	Organic
Housing Type^2^	Aviary	Aviary	Aviary	Aviary	Aviary	Aviary
Outdoor area^2^	WG & outdoor range	WG & outdoor range	None	None	WG & outdoor range	WG & outdoor range
Hen Breed	Dekalb White	Dekalb White	Lohmann Selected Leghorn	Dekalb White	Lohmann Brown	Dekalb White
Approximate number of hens	27,000	27,000	40,000	13,000	19,000	18,000
Bird density (hens/m^2^)	9	9	9	9	6	6
Age (weeks)	45	50	74	26	50	49
PRM present in house*	Y	N	Y	N	Y	N

1 Sampling locations are indicated by letter to distinguish farm type (A – Free range , B – Barn, C - Organic) and number to distinguish different poultry houses of the same type (I, II). A-I , A-II and B-II were located on the same farm, A-I and A-II are the same house at different time points.

2 All poultry houses had a similar indoor (aviary) housing system and a standard laying hen feed, except for C-I and C-II which received organic feed. The free range and organic houses had a WG (winter garden, a covered outdoor range) connected to the indoor barn and an uncovered outdoor range, with a larger outdoor pasture for the organic farms.

⁎ : Presence of poultry red mites was assessed by using AVIVET® Poultry Red Mite traps (Lammers et al., 2017).

Approximately 1.0 g of PRM (when present) and litter were collected from each poultry house in 30-mL polystyrene containers (Sterilin^TM^, Thermo Scientific, Bleiswijk, the Netherlands). Sample containers were weighed before and after sample collection. Prior to use, all polystyrene containers were baked-out for 3 days at 60°C in a Heratherm™ oven (Thermo Fisher, Amsterdam, the Netherlands) to minimize any VOC artefacts originating from the container material. Samples were collected in quadruplicate, processed on the day of collection and always kept at room temperature.

### Headspace sampling of volatile organic compounds

**Fig. 1:**
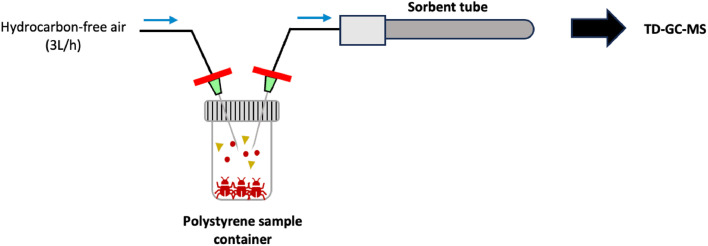
Schematic representation of the headspace sampling setup used for collection of VOCs from poultry red mite and litter.

Sample containers were placed in a holder and pierced with two 21 G stainless steel needles (BD Microlance^TM^, Fisher Scientific, Bleiswijk, the Netherlands) which served respectively as inlet and outlet of the purge gas. In- and outlet gas lines (1/8″ perfluoroalkoxy alkane) were equipped with Sartorius Minisart™ NML SFCA 0.2 µm syringe filters (Fisher Scientific, Bleiswijk, the Netherlands) to prevent contamination of the gas lines and sorbent tubes. Hydrocarbon-free air was generated through a catalyzer (SensorSense B.V., Nijmegen, the Netherlands) and served as purge gas. Prior to VOC collection, sample containers were purged for 1 min at 50 mL/min. Subsequently, the headspace of the sample containers was purged at a rate of 50 mL/min for 30 min to transfer produced VOCs onto Biomonitoring sorbent tubes (Markes International, Bridgend, UK). Empty polystyrene containers served as blank. A schematic overview of the headspace sampling setup is shown in Fig. 1.

### Thermal desorption – gas chromatography – mass spectrometry

Analyses of collected VOCs were carried out on a GC-MS QP2010 Ultra (Shimadzu, Kyoto, Japan) equipped with a TD-20 thermal desorption unit (Shimadzu, Kyoto, Japan). The desorption temperature was set to 260°C and sorbent tubes were desorbed for 8 min under a 60 mL/min-flow of helium (6.0 Scientific, Linde Gas, Dieren, the Netherlands). Desorbed VOCs were recollected on a Tenax TA focusing trap (Shimadzu, Kyoto, Japan) which was kept at –20°C. After, the focusing trap was rapidly heated to 250°C for 5 min to release the VOCs which were then transferred onto a CP-Sil 19 CF capillary column (length: 25 m, internal diameter: 0.25 mm, film thickness: 1.2 µm; Agilent Technologies, Amstelveen, the Netherlands) at a split ratio of 5:1. The carrier gas was helium and the flow was set to 1.0 mL/min. The column temperature was initially set at 35°C and increased to a final temperature of 250°C at a rate of 5°C/min. The final temperature was held for 5 min. Prior to use, all sorbent tubes were conditioned at 280°C for 2 h under a stream of N_2_ (100 mL/min) using a TC-20 sorbent tube conditioning station (Markes International, Bridgend, UK). Quantification was done using external calibration with toluene-d8 (99.6 atom % D; Sigma-Aldrich, Amsterdam, the Netherlands). Concentrations were corrected for sample weight.

### Reference standards

Heptanal (95 %), octanal (95 %), pyrrole (98 %), and 1-octen-3one (96 %) were purchased from Sigma-Aldrich (Amsterdam, the Netherlands). A stock solution was prepared in methanol (LiChromSolv®, VWR, Amsterdam, the Netherlands) containing 0.81 ng/μL heptanal, 0.82 ng/μL octanal, 0.97 ng/μL pyrrole, and 0.83 ng/μL 1-octen-3-one. 10 µL stock solution was spiked onto 5 Biomonitoring sorbent tubes and analyzed with TD-GC-MS using the same parameters as described above.

### Data analysis

Raw data were first converted to netCDF format in GCMS Solutions v.2.72 (Shimadzu, Kyoto, Japan). Subsequent processing of chromatograms was performed using PARADISe v6.0.1. (Johnsen et al., 2017; Quintanilla-Casas et al., 2023), a deconvolution and identification system for processing GC-MS data which employs a PARAFAC2 algorithm. Intervals where PARADISe applies deconvolution were selected manually along the full chromatogram. Modeling options were set to default (8 components per interval and 50,000 max iterations per interval). Models for each interval were selected to optimize for model fitting (> 95 %), model consistency, background removal, and avoiding overfitting. Identification of molecules was achieved using a NIST23 Mass Spectral Library (Shimadzu, Kyoto, Japan) and cross-checked against the raw mass spectra. Match factors were used to assign identities to the measured spectra. These represent a measure of spectral similarity and range from 0 to 999. Match factors greater than 800 are referred to a good match and match factors greater than 900 are considered an excellent match (Amirav et al., 2025). Here, the threshold for identification was set to 800. The resulting peak area matrix was exported in .xlsx format and processed using an in-house MATLAB (R2020a, The Mathworks Inc., Natick, MA) script. Missing values were replaced with 1/5 of the lowest feature value (Das et al., 2022) and subsequently autoscaled.

After processing, the aligned peak matrix was subjected to principal component analysis **(PCA).** Partial least squares-discriminant analysis (**PLS-DA**) was used to distinguish the differences in VOC profiles between litter and mite samples for mite-infested farms. VOCs for univariate statistical comparison were selected based on the variable importance on projection (**VIP**) values of the PLS-DA model (VIP > 1). Known artefacts (e.g. column bleed) were removed from the data. Univariate statistical comparison of peak areas was done using a Mann-Whitney test, followed by a Benjamini-Hochberg post-hoc correction. Target VOCs were cross-checked in both positive and negative control samples. VOC production rates per gram of sample for PRM samples, litter samples from PRM-infested farms (positive control) and litter samples from PRM-free farms (negative control) were statistically compared using a Kruskal-Wallis test. Results were analysed using Dunn's test and a P-value of less than 0.05 was considered significant.

## Results and discussion

### Confirmation of poultry red mite infestation

Although quantification of PRM using cardboard traps such as the AVIVET® trap is generally assumed to be more sensitive to low PRM numbers than visual scoring using MMS (Mul et al., 2015; Oh et al., 2020), this method cannot be used to determine total absence of PRM with complete certainty (Lammers et al., 2017). Therefore, poultry houses were considered free of PRM and regarded as negative control only when no PRM were found with both trapping and visual methods.

For each poultry house, Table 2 shows the average PRM weight across all traps. The results show varying average PRM infestation levels in the positive control farms (A-I, B-I and C-I) and confirm the absence of the PRM in the negative control farms (A-II, B-II and C-II).

**Table 2:** Overview of poultry red mite population assessment across poultry houses on laying hen farms.

	Farm					
Farm code	A-I	A-II	B-I	B-II	C-I	C-II
Number of traps placed	20	20	20	10	10	10
Average mite weight (mg)	22.1	0	1922	0	51.9	0

### The odor profile of the poultry red mite

For deconvolution of the chromatograms by PARADISe, 153 intervals were manually selected. In total, 229 peaks were found, and 172 remained after removing artefacts. For 120 peaks, an identity could be assigned through the NIST23 mass spectral library (match factor > 800).

**Fig. 2:**
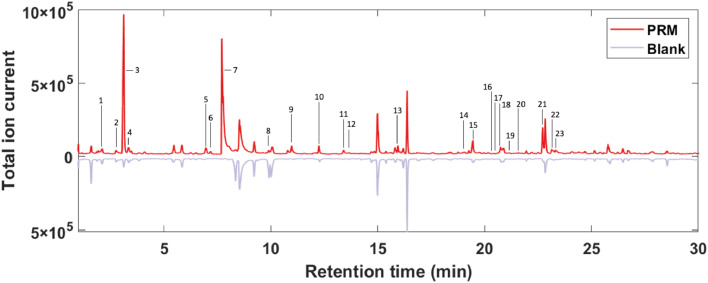
Median chromatograms for poultry red mite samples (top) and blank polystyrene containers. Characteristic peaks for PRM are indicated at their specific retention time.

For first exploration of the VOC profiles, PRM chromatograms were compared with chromatograms obtained from blank Sterilin™ containers (Fig. 2). Indicative peaks for PRM were identified by selecting peak areas greater than the peak area of the blank plus 3 standard deviations. This resulted in a list of 23 characteristic VOCs related to PRM (Table 3).

**Table 3:** Poultry red mite odor profile molecules.

Peak #	Compound	CAS #	Retention time (min)	Average VOC production (μg *g*^−1^ h^−1^)	Odor Character^1^
1	Trimethylamine	75-50-3	1.83	1.24 ± 1.10	Fishlike to ammonia-like
2	Dimethyl sulfide	75-18-3	2.89	0.25 ± 0.41	Rotten vegetable^2^
3	Acetone	67-64-1	3.07	234 ± 214	Characteristic. Sweet, minty; like nail polish
4	Isopropyl alcohol	67-63-0	3.33	9.29 ± 8.04	Sharp, musty, like rubbing alcohol
5	3-methylbutanal	590-86-3	6.97	4.95 ± 4.02	Pungent, fruity, malt-like, almond-like
6	2-methylbutanal	96-17-3	7.17	2.41 ± 1.41	Malt-like, cacao, apple^3^
7	1-Vinylaziridine	5628-99-9	7.68	146 ± 193	Not reported in literature
8	Dimethyl disulfide	624-92-0	9.57	0.21 ± 0.14	Strong, nauseating, garlic-like
9	Acetoin	513-86-0	10.93	9.41 ± 11.8	Buttery; bland, woody, yoghurt
10	Hexanal	66-25-1	12.19	16.7 ± 21.0	Sharp, fruity, grass-like
11	Pyrrole	109-97-7	13.33	5.51 ± 6.04	Agreeable; chloroform
12	Chlorobenzene	108-90-7	13.34	0.18 ± 0.20	Sweet, almond-like
13	Heptanal	111-71-7	15.87	20.0 ± 3.35	Strong, fruity
14	1-Octen-3-one	4312-99-6	18.67	1.17 ± 1.57	Metallic; mushroom-like^4^
15	Octanal	124-13-0	19.38	9.84 ± 1.10	Strong, fruity; orange-like
16	1-(2-methoxy-1-methylethoxy)-2-propanol	20324-32-7	20.62	1.28 ± 0.41	Odorless^5^
17	2-ethyl-1-hexanol	104-76-7	20.76	2.62 ± 2.67	Sweet, slightly floral; characteristic
18	Decamethyl cyclopentasiloxane	541-02-6	20.85	7.00 ± 8.04	Odorless
19	Unidentified	N/A	21.17	0.20 ± 0.26	N/A
20	Hexanoic acid	142-62-1	21.3	0.26 ± 0.18	Unpleasant; goat-like; cheese-like
21	Nonanal	124-19-6	22.65	23.3 ± 34.4	Floral; orange, rose
22	Acetophenone	98-86-2	23.12	3.36 ± 2.29	Sweet, medicinal; orange-like
23	Phenol	108-95-2	23.31	1.67 ± 1.25	Sweet, acrid, sickening; tar-like

1 (National Library of Medicine, 2024).

2 (Suffet and Rosenfeld, 2007).

3 (Chen et al., 2020).

4 (Lubran et al., 2005).

5 (Kimya, 2020).

Fig. 2 The PRM odor profile comprises 22 identified volatiles that were enhanced in mites across 3 different common poultry farming systems (barn, free range and organic). One VOC remained unidentified (Supplementary Figure S1). To the best of our knowledge, no previous research has been conducted on the odor profile of PRM. Although multiple studies have been done on the behavioral reaction of the PRM to several odorous compounds (Koenraadt and Dicke, 2010; Kim et al., 2016; Pavela and Benelli, 2016; Lee et al., 2019; El Adouzi et al., 2020; Gay et al., 2020; Auffray et al., 2023), the VOCs from the PRM itself have not previously been studied.

Comparing the VOCs of Table 3 to the expert description of the PRM odor as “sweetish, musty and metallic”, several components may be responsible for this distinct scent. Interestingly, the VOC 1-octen-3-one is specifically described to have a “metallic” odor character. The “musty” and “sweet” odor notes can be attributed to several VOCs in the list; 13 out of 23 are described to have a sweet, fruity or musty odor character. Six out of these 13 VOCs are aldehydes ((3-methylbutanal, 2-methylbutanal, hexanal, heptanal, octanal, and nonanal). As aldehydes are known for their fragrant properties and used to enhance a wide range of odor notes in applications such as perfume development (Kim et al., 2019), and several aldehydes have previously been identified as pheromones of other hematophagous parasites (Siljander et al., 2008; Kilpinen et al., 2012), it is likely that the sweet odor notes could stem from these VOCs.

Not all identified VOCs may be part of the actual odor profile of the PRM: some could derive from the aviary structure instead of from the PRM. Due to the PRM collection method, it is likely that dust from the housing system will have been brushed into the vials along with the PRM. This dust will contain VOCs related to poultry production odors in general. This influence of VOCs from the environment of the PRM is inherent to the design of the current study, where samples collected directly from the field are studied. Experiments under controlled laboratory conditions could yield valuable insights into the PRM volatilome, without such interference from the housing system. Under controlled conditions, also the effect of circumstances such as fed versus starved and PRM developmental stage and sex on the PRM odor profile could be studied.

Known classes of such potentially interfering odorants from poultry production facilities are volatile fatty acids, volatile amines, phenols and sulfur-containing compounds (Lacey et al., 2004). The following VOCs from the PRM profile shown in Fig. 2 and Table 3 are part of these classes: hexanoic acid, trimethylamine, phenol, dimethyl sulfide, and dimethyl disulfide. Further, acetoin was found to be an important predictor of odor from tunnel ventilated broiler sheds (Murphy et al., 2014). Specifically for laying hen farm odor emissions, one study found VOCs corresponding to 8 VOCs identified here from the mite odor profile: phenol, dimethyl sulfide, acetone, acetophenone, 2-ethyl-1-hexanol, acetoin (3‑hydroxy-2-butanone), 3-methylbutanal and nonanal (Tanaka et al., 2020). It is therefore likely that these VOCs stem from dust from the housing structure, instead of from the PRM themselves.

Silicate-based products are used in many farms as a control measure against the PRM (Hwang, 2023). This may explain the presence of the decamethylcyclopentasiloxane in the odor profile. In addition, the unidentified compound shows a characteristic siloxane mass spectrum, which could originate from the same source. Other possible non-PRM sources of VOCs in the odor profile may be remnants from cleaning materials or pesticides. This could explain the presence of chlorobenzene, 1-(2‑methoxy-1-methylethoxy)-2-propanol and isopropyl alcohol. These findings are relevant for future field studies about the application of odor-based detection methods to monitor animal welfare in laying hen farms in general.

Both 1-vinylaziridine and pyrrole were not previously reported as VOCs from acari or poultry odor emissions. Further investigation is required for their origin as a VOC in the PRM odor profile.

Apart from the various possible non-mite sources of VOCs identified in the PRM odor profile, several VOCs remain that likely stem from the PRM themselves. 1-Octen-3-one seems especially feasible, as the odor character matches the expert description of the PRM scent. Possibly the sweet and musty scent components are linked to one or more of the identified aldehydes.

### Multivariate analysis

**Fig. 3:**
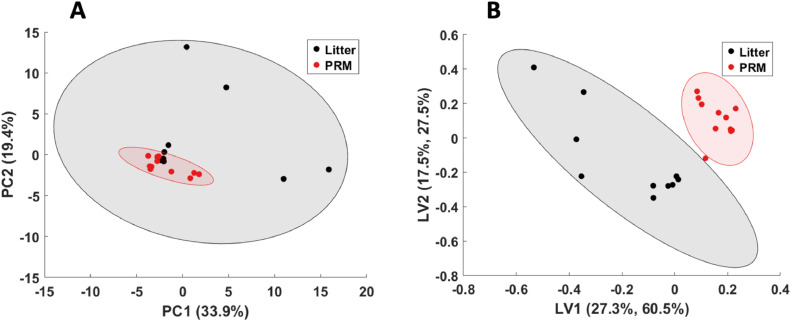
Multivariate analysis of poultry red mite and litter headspace VOC profiles. (A) PCA scores plot for the first and second principal component, (B) PLS-DA scores plot for the first and second latent variable.

Following the exploration of the PRM VOC profile by comparison to empty collection vials, further analysis was performed to identify targets for PRM detection across different housing systems. First, processed data from PRM and litter samples obtained at positive control farms were subjected to principal component analysis for initial exploration of the VOC profiles (Fig. 3A). Litter samples were used as contrast to the PRM samples, as litter is known from literature to be the most odor-determining compound in poultry farms (Dunlop et al., 2016) and was therefore considered representative for the background odor of the poultry farms. Odor compounds from the sample vials were not considered in the analysis.

It was observed that the scores for the first and second principal component overlap, indicating that the majority of the variance in the data is not driven by PRM-specific VOCs. In addition, the litter VOC profiles display large differences. This variance between samples can be attributed to farm effects, such as differences in litter composition, management strategies, and feed.

To aid in the discrimination of PRM and litter data, PLS-DA was used (Fig. 3B). As PLS is a supervised technique, proper care has to be taken that the model is not overfitted, hence validation of the model has to be performed (Westerhuis et al., 2008). As the data set is relatively small, leave-one-out cross validation was used to estimate the model performance (Godlewski et al., 2023) and a permutation test was subsequently run to assess whether the model performs better than a random PLS model (Westerhuis et al., 2008). The cross-validation resulted in an excellent goodness-of-prediction (Q^2^) value of 0.780 (the empirically inferred acceptable value for biological models is > 0.4 (Worley and Powers, 2013)). The permutation test revealed that the original PLS-DA model outperforms any random model (*P* < 0.001). This showed that the PRM VOC profiles can be discriminated from litter VOC profiles independent of housing system.

The next step was to identify which PRM-related VOCs are responsible for the discrimination from litter. VIP scores were calculated and scores greater than 1 were considered to have discriminatory power. Univariate statistical analysis (Mann-Whitney test with Benjamini-Hochberg post-hoc correction) was applied to find the compounds that were statistically significantly enhanced in either sample.

Based on the screening criteria VIP > 1 and *P* < 0.05, 12 VOCs were included that could be targets for mite presence or absence across different commercial poultry farming systems. Of these 12 VOCs, 5 were more abundant in PRM samples whilst the remaining 7 VOCs displayed lower abundance in PRM VOC profiles (Table 4). The identity of VOCs enhanced in PRM headspace was confirmed, when available, with reference standards (Supplementary Table S2), as these VOCs can serve as potential targets for PRM detection and monitoring.

**Table 4:** Significant VOCs for PRM and litter samples collected at positive control farms.

Compound	Average retention time (min)	Log_2_ fold change	P-value
Higher abundance in PRM samples			
1-Vinylaziridine	7.68	6.87	0.009
Heptanal	15.87	3.19	0.034
Pyrrole	13.33	2.85	0.009
1-Octen-3-one	18.7	2.16	0.036
Octanal	19.38	1.27	0.034
Lower abundance in PRM samples			
2,4-Pentadienenitrile	12.14	−4.42	0.030
Dimethyl disulfide	9.57	−4.04	0.030
3-Methyl-2-pentanone	10.53	−3.27	0.030
2-Methylfuran	4.73	−3.23	0.036
2-Methylhexane	4.89	−1.58	0.034
5-Methyl-3-hexen-2-one	7.44	−1.58	0.034
2-Pentanone	7.34	−1.11	0.034

The VOCs 1-vinylaziridine, heptanal, 1-octen-3-one, octanal, and pyrrole (all identified in the odor profile of the PRM in Fig. 2 and Table 3) were enhanced (*P* < 0.05) in PRM samples. Seven molecules were enhanced (*P* < 0.05) in litter headspace. Of these molecules, dimethyl disulfide also occurred in the odor profile of the mite when contrasted with blank vials. As discussed previously, this is likely due to the collection method, where dust from the system could have been collected along with PRM. The other 6 litter molecules did not occur in the PRM odor profile and likely originate from the litter, feces or the hens themselves. The VOC 2-pentanone was previously identified as a VOC in laying hen farm odor emissions (Tanaka et al., 2020). For the remainder of the VOCs with lower abundance in PRM samples, no report was found on poultry odor emission litter. However, 2,4-pentadienenitrile, 2-methylfuran and 2-methylhexane were previously identified in swine manure and could potentially stem from feces (Schiffman et al., 2001). For the remaining litter-specific VOCs (3-methyl-2-pentanone and 5-methyl-hexen-2-one), the origin is currently unknown.

**Fig. 4:**
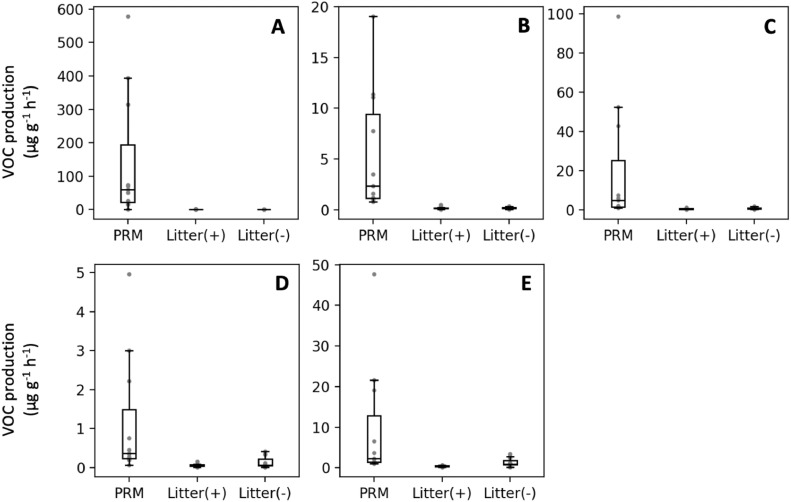
Calculated VOC emission rates in μg VOC per gram of sample per hour of PRM target VOCs for PRM samples (PRM) and litter samples from negative (Litter(-)) and positive control farms (Litter(+)). Target VOC: (A) 1-vinylaziridine (B) pyrrole; (C) heptanal; (D) 1-octen-3-one; (E) octanal.

To validate the target VOCs for PRM detection, odor emissions per gram of PRM were compared against litter from both negative (PRM-free) and positive control (PRM-infested) farms. Fig. 4 shows that emissions of target VOCs are lower in all litter samples compared to PRM samples (*P* < 0.05 for all VOC targets). All 5 targets were found to be highly specific for PRM as these VOCs were emitted at higher concentrations (*P* < 0.05) compared to litter from both positive and negative control farms.

Target VOC emissions were not lower (*P* > 0.05) in litter from negative control farms compared to positive control farms, indicating that PRM were likely not present in the litter samples even in infested farms and that litter samples give an adequate representation of background odor.

It appears that the target VOCs are enhanced in PRM-infested farms independent of housing type, indicating that these are promising VOCs for PRM infestation detection. As the VOC profile in a poultry facility can vary throughout the year (Pillai et al., 2012; Huang and Guo, 2020), further research is required to investigate how seasonal influence could affect odor-based PRM detection.

The highest production rates were reported for 1-vinylaziridine (Fig. 4A) and heptanal (Fig. 4C). Pyrrole (Fig. 4B), octanal (Fig. 4E) and especially 1-octen-3-one (Fig. 4D) had low production rates. It may be difficult in practice to target these molecules for early detection of the PRM as the concentrations may be below the detection limit when a low amount of PRM is present in the poultry house.

The odor characteristics of several PRM VOC targets match the odor description provided by experts. For instance, 1-octen-3-one was described as a compound with a distinctly ‘metallic’ odor and both heptanal and octanal are aldehydes with fruit-like, sweet odor character. Further studies could corroborate whether the VOC targets match the scent of the PRM performing gas chromatography coupled with mass spectrometry and olfactometry (GC-MS-O) and an odor assessment panel of poultry experts in the odor of a PRM infestation.

In an earlier study, 1-octen-3-one was identified as a key odorant in the metallic scent of blood on skin (Glindemann et al., 2006). As the PRM is an obligate blood-feeder, this may explain that 1-octen-3-one was found as a volatile biomarker. For other hematophagous parasites, 1-octen-3-one was also reported as a VOC in the feces of head lice (*Pediculus humanus capitis*) (Galassi et al., 2020) and found to be produced by insects from the blood-feeding genus *Triatoma*. (May-Concha et al., 2015, 2018).

Hexanal and octanal have not previously been linked to VOC profiles in acari or other blood-feeding parasites. However, prior research on bed bugs (*Cimex lectularius*) have shown that several aldehydes are produced by this species as alarm or aggregation pheromone (Schildknecht et al., 1964; Siljander et al., 2008; Kilpinen et al., 2012; Dery et al., 2020). PRM and *C. lectularius* show similar behavior in that they are obligate blood-feeders that feed at night and aggregate in refuges during the day. Aldehydes could potentially have a similar function in the PRM as in *C. lectularius*.

1-vinylaziridine was most enhanced (highest log-fold change) across PRM samples compared to litter samples. Therefore, it can be suggested that 1-vinylaziridine is important for distinguishing PRM odor from litter background. The origin of this VOC is not readily explained in literature. It has not been identified in previous studies as a VOC in other mite species or blood-feeding parasites. It has only been reported earlier as a volatile biomarker for *Pseudomonas aeruginosa* (Filipiak et al., 2012; Chingin et al., 2015).

On the aviary systems, PRM cluster together and do not appear to move during the daytime. During collection of such clusters into vials and subsequent laboratory analysis, the mites were disturbed and became active, moving through the sampling tube and occasionally clustering together into small spherical groups. Although little is known about the biological activity of naturally occurring aziridine compounds, defensive properties such as antibacterial qualities have been reported (Lowden, 2006). Speculatively, the release of 1-vinylaziridine by the PRM may be linked to a defense mechanism or alarm response as a result of physical disturbance during sample collection.

Although the origin and function of this VOC cannot be ascertained, its specificity to all collected PRM samples and the fact that it is not a commonly found VOC in previous studies makes it especially interesting as a potential target for odor-based detection of the PRM.

1H-pyrrole was found as a VOC in diverse studies, ranging from bacteria to human cancer cells (Filipiak et al., 2010), but the biochemical or metabolic origin of its emission as VOC has not been determined. In bacteria, it has been found as a volatile biomarker for *Campylobacter* in poultry feces samples (Garner et al., 2008) and in bacterial cultures of *P. aeruginosa* (Filipiak et al., 2012) and *Klebsiella pneumonia* (Hintzen et al., 2024). This molecule has not previously been described as a volatile marker of acari species or insect parasites.

1H-Pyrrole could be a product of metabolic processes of *D. gallinae* or its microbial symbionts, however, it has not been reported as a VOC from other blood-feeding species or linked to the catabolism of blood or heme in such parasites (Paiva-Silva et al., 2006; Toh et al., 2010; Sojka et al., 2013). The pyrrole group is a key part of many biologically active molecules (Walsh et al., 2006; Domagala et al., 2015). As heme contains 4 pyrrole moieties, it could serve as a source of free pyrrole, or it could originate from the digestion of other biological molecules such as proteins. The metabolic pathways of blood digestion in *D. gallinae* have not yet been elucidated, although transcriptome analysis did show differences between blood-fed and starved PRM (Fujisawa et al., 2020). One study also demonstrated the importance of the microbial community in the gut of *D. gallinae* in its digestive processes (Liu et al., 2024). It is clear that these digestive pathways are complex and the relation to VOC emission is not well understood. Further, different species may have evolved specialized pathways resulting in different volatile end products (Maoz et al., 2022).

In conclusion, the odor profile of the PRM was established for the first time using TD-GC-MS and multivariate analysis. This resulted in 23 VOCs from which 5 VOC targets (1-octen-3-one, heptanal, octanal, 1-vinyl-aziridine, and 1H-pyrrole) are promising markers for PRM detection independent of housing system. It shows that regardless of factors such as feed and farm management, PRM could be unambiguously identified using VOC analysis. By using the experts’ description of PRM scent, the VOCs 1-octen-3-one, heptanal and octanal were linked to the PRM odor production, whilst the origin of the other targets 1-vinyl-aziridine and 1H-pyrrole remains unclear. This opens new avenues towards developing a selective system for VOC-based monitoring of the PRM population, research on how these molecules should be measured, and further investigation (e.g. PRM metabolome studies) of the origin of PRM-specific VOCs.

## Declaration of competing interest

The authors declare the following financial interests/personal relationships which may be considered as potential competing interests:

Joris Meurs reports financial support was provided by Dutch Research Council. Simona Cristescu reports financial support was provided by Dutch Research Council. Francisca Velkers reports financial support was provided by Dutch Research Council. If there are other authors, they declare that they have no known competing financial interests or personal relationships that could have appeared to influence the work reported in this paper.

## Appendix Supplementary materials

Image, application 1
