# Supplementary material for: Odor characterization of the poultry red mite (Dermanyssus gallinae) for identification of volatile biomarkers of infestation across multiple commercial laying hen systems
Source: Poult Sci. 2025 Mar 25;104(6):105101. doi: 10.1016/j.psj.2025.105101 (PMC11997325; doi:10.1016/j.psj.2025.105101)
Supplement: Supplementary file 1 [file mmc1.docx]

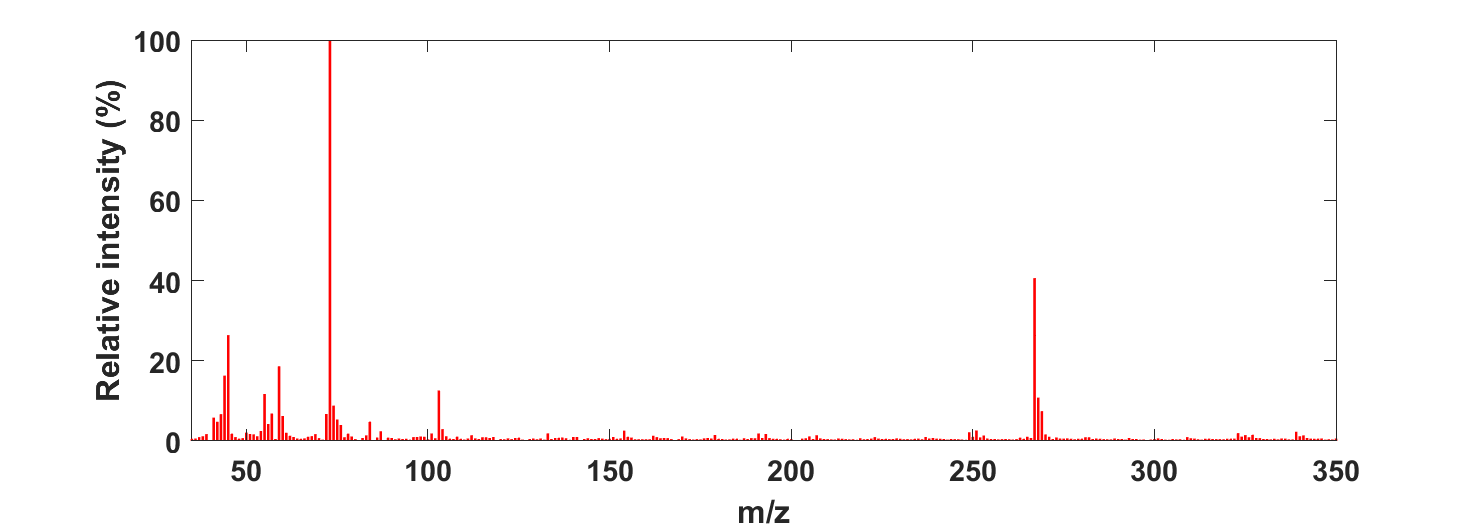
Figure S1: Mass spectrum of unidentified VOC from PRM odor profile

Table S2: Confirmation of identities for target volatile organic compounds in relation to PRM odor

| **Compound** | **Chromatogram** | **Mass spectrum** | **Similarity index** |
| --- | --- | --- | --- |
| Pyrrole | 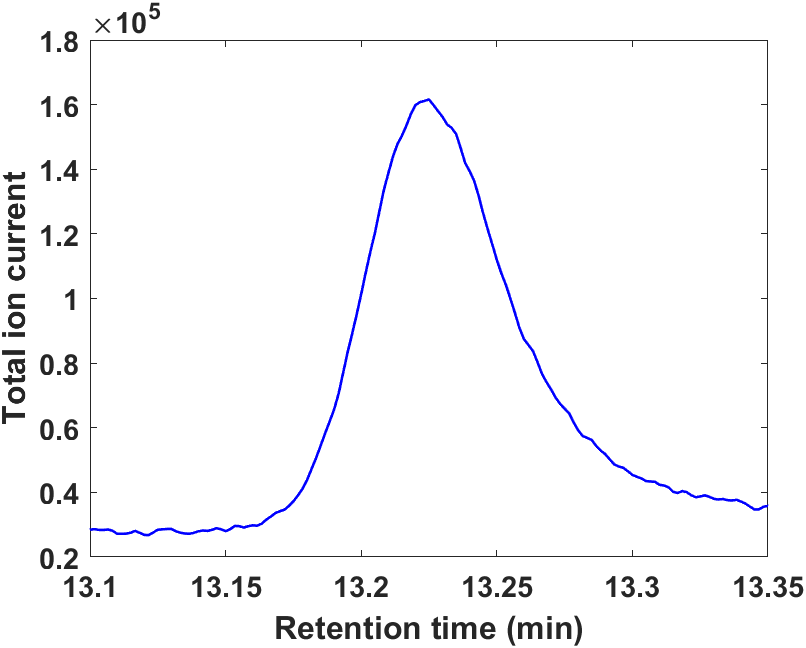 | 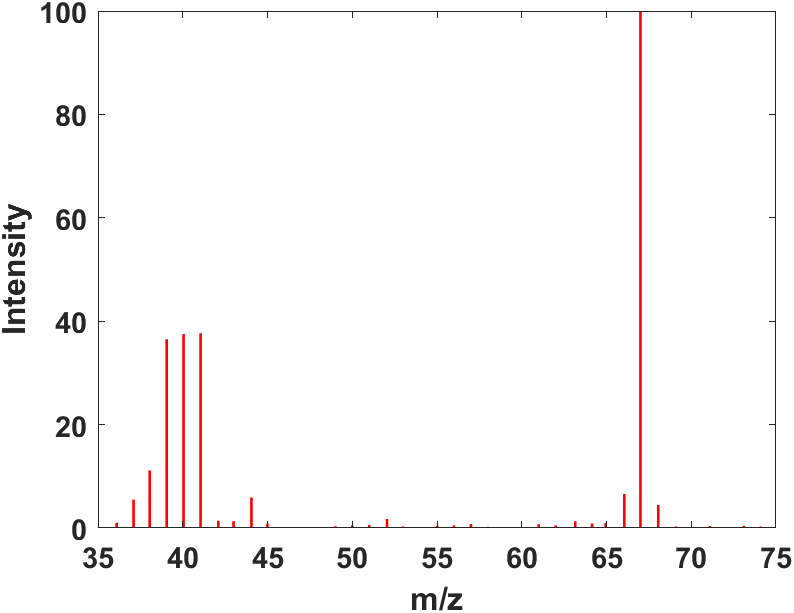 | 90 |
| Heptanal | 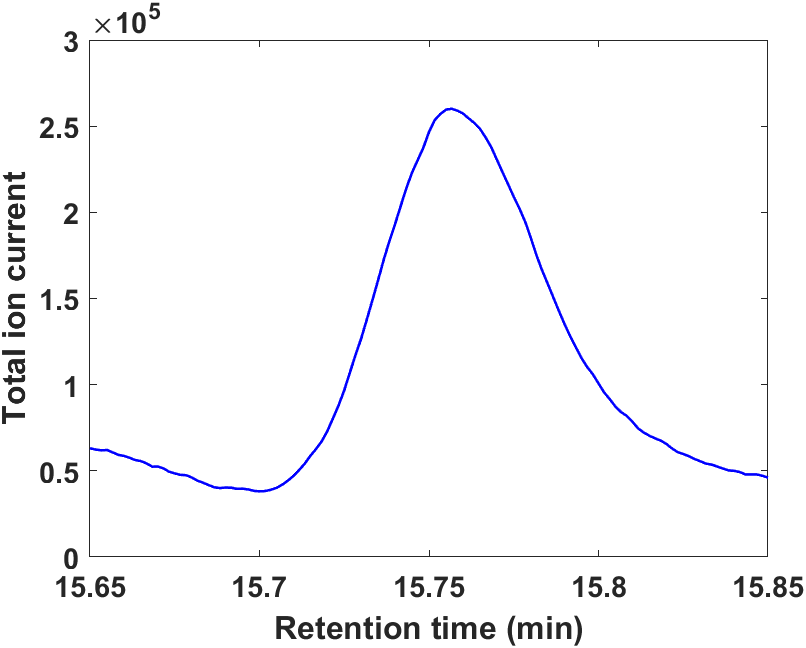 | 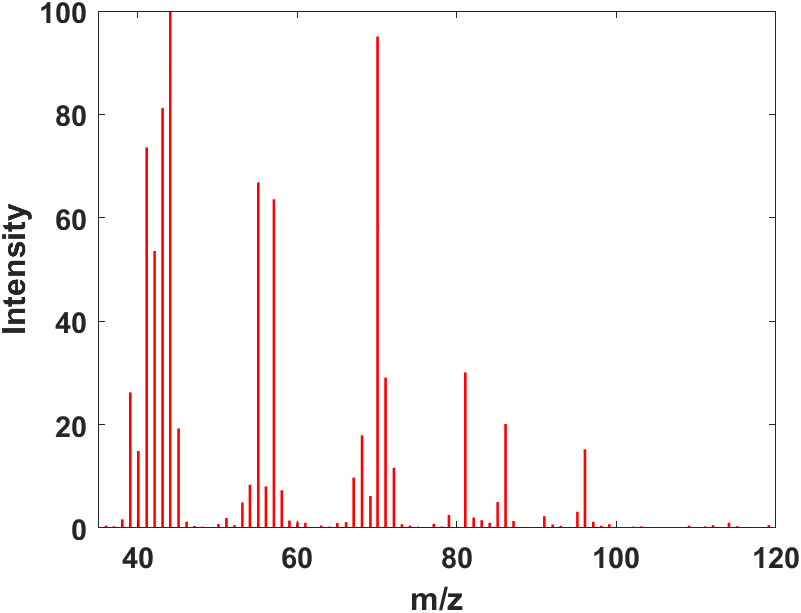 | 97 |
| 1-Octen-3-one | 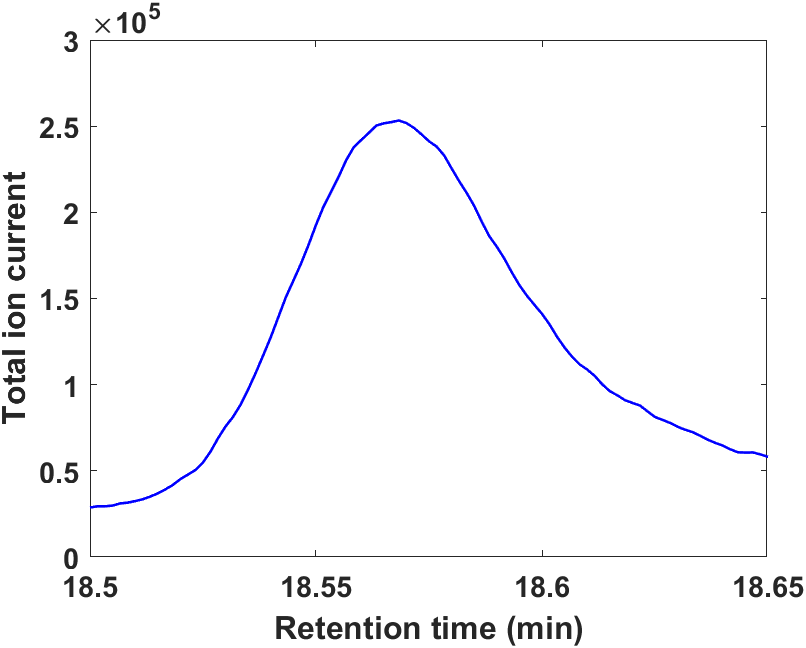 | 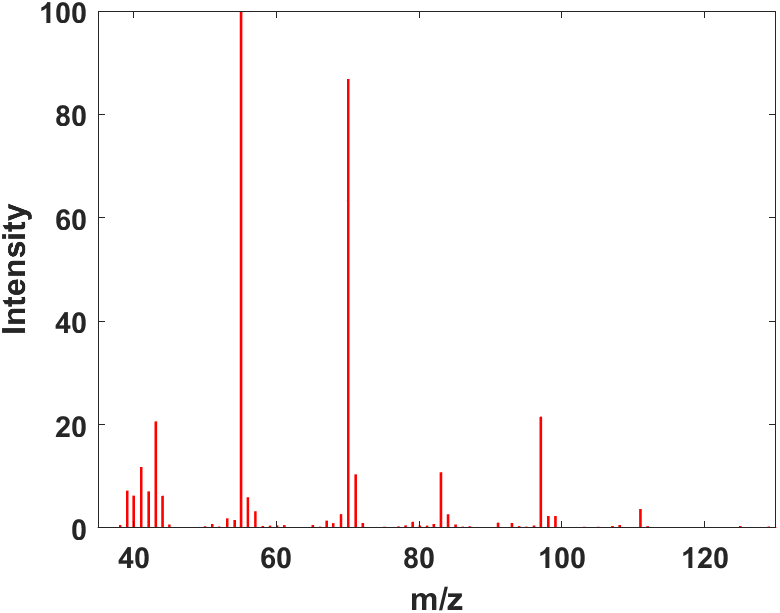 | 94 |
| Octanal | 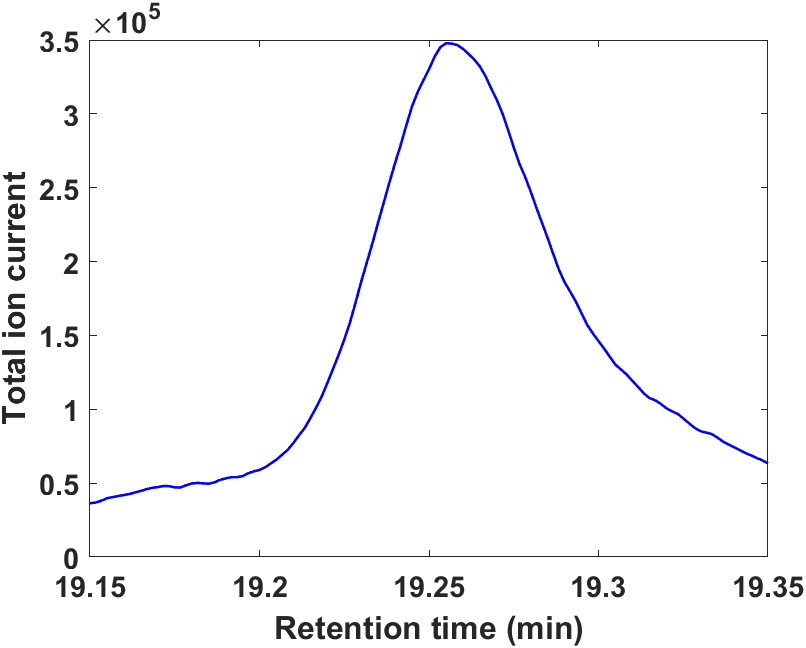 | 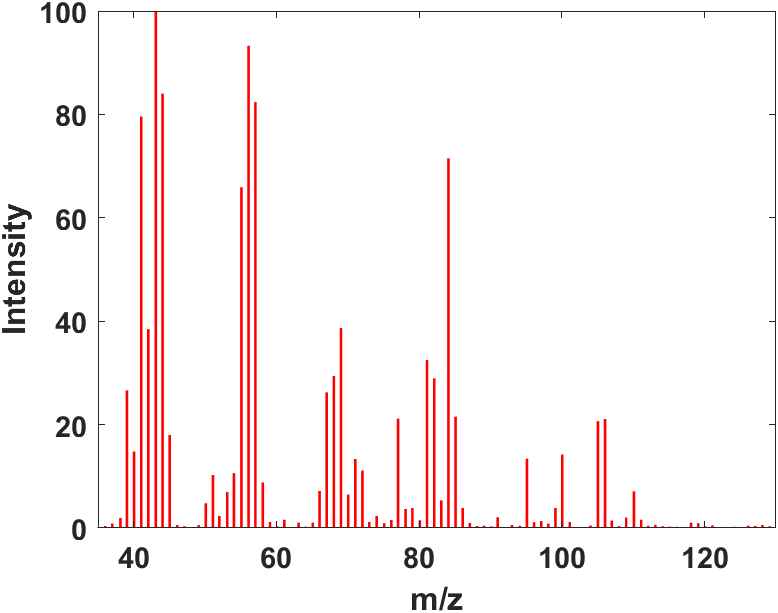 | 90 |

No reference standard available for 1-vinylaziridine.
